# circFAM160A2 Promotes Mitochondrial Stabilization and Apoptosis Reduction in Osteoarthritis Chondrocytes by Targeting miR-505-3p and SIRT3

**DOI:** 10.1155/2021/5712280

**Published:** 2021-10-04

**Authors:** Jiapeng Bao, Changjian Lin, Xing Zhou, Diana Ma, Lujie Ge, Kai Xu, Safwat Adel Abdo Moqbel, Yuzhe He, Chiyuan Ma, Jisheng Ran, Lidong Wu

**Affiliations:** ^1^Department of Orthopedic Surgery, The Second Affiliated Hospital, Zhejiang University School of Medicine, Hangzhou City, Zhejiang Province, China; ^2^Orthopedics Research Institute of Zhejiang University, Hangzhou City, Zhejiang Province, China; ^3^Key Laboratory of Motor System Disease Research and Precision Therapy of Zhejiang Province, Hangzhou City, Zhejiang Province, China

## Abstract

Competitive endogenous RNAs (ceRNAs), as a newly identified regulating mechanism, have been demonstrated to play a crucial role in various human diseases. An increasing number of recent studies have revealed that circular RNAs (circRNAs) can function as ceRNAs. However, little is known about the role of circFAM160A2 in the pathological process of osteoarthritis (OA). This study is the first to examine the crucial role of the circFAM160A2-miR-505-3p-SIRT3 axis in osteoarthritis progression. miR-505-3p was selected from the interaction of a microRNA (miRNA) microarray comparing chondrocytes in OA and normal conditions and prediction results from TargetScan. RT-qPCR was performed to assess the expression of circFAM160A2, miR-505-3p, and SIRT3. A dual luciferase assay was used to validate the binding of circFAM160A2, miR-505-3p, and SIRT3. We used lentivirus and adeno-associated virus to establish in vitro and in vivo overexpression models. Western blotting, apoptosis assay, ROS detection assay, Safranin O staining, and CCK-8 assay were employed to assess the role of circFAM160A2, miR-505-3p, and SIRT3. We found that miR-505-3p was upregulated and circFAM160A2 was downregulated in OA. While overexpression of circFAM160A2 decreased the production of extracellular matrix (ECM) degrading enzymes and ameliorated chondrocyte apoptosis and mitochondrial dysfunction, inhibition of miR-505-3p could reverse the protective effect of circFAM160A2 on the OA phenotype both in vitro and in vivo. In conclusion, circFAM160A2 can promote mitochondrial stabilization and apoptosis reduction in OA chondrocytes by targeting miR-505-3p and SIRT3, which might be a potential therapeutic target for OA therapy.

## 1. Introduction

Osteoarthritis (OA) is the most common joint disorder, leading to a massive socioeconomic cost as well as causing pain or even disability to the estimated 10% of men and 18% of women over 60 years of age affected worldwide [1, 2]. Researchers have reported that OA is characterized by progressive articular cartilage loss, osteophyte formation, synovial inflammation [3], and impairment of mitochondrial function [4]. Nevertheless, treatment is limited to pain management or joint replacement for end-stage disease [5], as the underlying molecular mechanism driving OA pathology remains elusive.

Mitochondrial dysfunction is a hallmark of OA and has recently drawn particular attention [6]. The sirtuin (SIRT) family, a class of evolutionarily conserved NAD^+^-dependent deacetylase proteins, contributes to the regulation of energy balance [7]. Recent insights have revealed that among the sirtuin family members, SIRT3 plays a chondroprotective role in OA by maintaining mitochondrial homeostasis [8] and preserving mitochondrial DNA integrity and function [9]. However, the upstream regulatory mechanism of SIRT3 has not yet been fully elucidated.

Circular RNAs (circRNAs) are a special subclass of endogenous noncoding RNAs produced by a noncanonical splicing event called backsplicing [10]. They are highly stable because of their covalently closed ring structure, and some of them are evolutionarily conserved [11]. Furthermore, previous studies reported that circRNAs can exert their biological function as microRNA (miRNA) sponges [12, 13] and RNA-binding protein sponges [14] and more controversially, through protein translation [15]. The emerging roles of RNA-RNA crosstalk are further confirmed as our knowledge of noncoding RNAs has expanded. However, little is known about the functional regulation of competing endogenous RNAs on SIRT3 in OA.

In this study, we aimed to discern the potential upstream regulatory mechanism of SIRT3 in OA pathological progression using RNA deep-sequencing technology and substantial experiments in vitro and in vivo.

## 2. Materials and Methods

### 2.1. Ethical Approval

All animal experiments were approved by the Ethics Committee of the Second Affiliated Hospital of Zhejiang University School of Medicine and carried out under the guidelines of the *Guide for the Care and Use of Laboratory Animals* published by the National Institutes of Health.

### 2.2. Clinical Samples

OA tissue samples and normal tissue samples from 5 OA patients and 5 femoral neck fracture patients, respectively, were collected from the surgical specimen archives of Second Affiliated Hospital of Zhejiang University School of Medicine, Zhejiang, China. All the procedures were approved by the Institutional Review Board of the Second Affiliated Hospital of Zhejiang University School of Medicine. We have obtained written informed consent from all study participants. All of the procedures were performed in accordance with the Declaration of Helsinki and relevant policies in China.

### 2.3. Reagents

Recombinant rat IL-1*β* was purchased from R&D Systems, Abingdon, UK. Dulbecco's modified Eagle's medium (DMEM), penicillin/streptomycin, fetal bovine serum (FBS), and 0.25% trypsin were obtained from Gibco BRL, Grand Island, NY, USA. Collagenase II was purchased from Sigma-Aldrich, St. Louis, MO, USA.

### 2.4. Luciferase Assay

To confirm the database binding prediction that the 568th to 575th bases of the SIRT3 3′ untranslated region (UTR) was specifically bound by miR-505-3p and miR-505-3p was specifically bound by circFAM160A2, the Dual Luciferase® Reporter Assay System (Promega, E1910, USA) was performed according to the manufacturer's instruction. Lipo3000 (Thermo Fisher, L3000150, USA) was used for transfection.

### 2.5. Lentiviral Overexpression Model

We used lentivirus to establish overexpression models of SIRT3, miR-505-3p, and circFAM160A2. The lentivirus circFAM160A2 was constructed and packaged by Hanbio (Shanghai, China). The lentivirus SIRT3 and miR-505-3p were constructed and packaged by (Genechem, Shanghai, China).

### 2.6. Bioinformatics Analysis

The miRNA targets of SIRT3 were predicted using the bioinformatics database TargetScan (http://www.targetscan.org/). We predicted the circRNA binding sites of miR-505-3p using the database miRanda (http://www.microrna.org). After selecting the results with a high level of evidence based on their indexes, we performed the overlapping interactions with a Venn diagram constructed by a web-based tool (http://bioinformatics.psb.ugent.be/webtools/Venn/).

### 2.7. Western Blotting

The adherent cells were washed three times with PBS and then detached with a cell scraper. Thereafter, the harvested cells were lysed in RIPA Lysis Buffer (P0013B, Beyotime, China) containing a protease inhibitor cocktail (P1005, Beyotime, China), a protein phosphatase inhibitor (P1260, Solarbio Science & Technology, Beijing, China), and a PMSF (A610425-0005, Sangon Biotech, Shanghai, China) on ice for 30 min. The Bradford Protein Assay Kit (P0006, Beyotime, China) was conducted according to the manufacturer's instruction to measure the protein concentrations. An aliquot of 15 *μ*g of total proteins from each sample was separated by SDS-PAGE gels at 100 V for 1.5 h and transferred onto polyvinylidene difluoride (PVDF) membranes (IPVH00010, Millipore, Billerica, MA, USA) at 300 mA for 1 h. Subsequently, the membranes were blocked for 2 h with 5% BSA (A600903, Sangon Biotech, Shanghai, China) at room temperature. After washing three times with TBST, the membranes were incubated with primary antibodies against GAPDH (1 : 1000, ab181602, Abcam), SIRT3 (1 : 500, ab118334, Abcam), MMP13 (1 : 1000, ab84594, Abcam), ADAMTS4 (1 : 500, ab185722, Abcam), collagen II (1 : 1000, NB600-844, RD), Bax (1 : 1000, ab32503, Abcam), Bcl-2 (1 : 500, ab194583, Abcam), and procaspase 3 (1 : 1000, ab90437, Abcam) overnight at 4°C. The next day, the membranes were washed three times with TBST and were incubated with horseradish peroxidase- (HRP-) conjugated goat anti-mouse and anti-mouse secondary antibodies (31160, 31210; 1 : 5000, Thermo Pierce) for 2 h. After washing three times with TBST again, the membranes were measured by using the SuperSignal® West Dura Extended Duration Substrate (34075, Thermo Fisher, USA). ImageJ software was performed to quantify the intensity of each band.

### 2.8. RNA Extraction and qRT-PCR

Total RNA was extracted using the Rapid Extraction kit for animal total RNA (GK3016, Generay Biotech, Hangzhou, China) while miRNA isolation was performed by the PureLink® miRNA Isolation Kit (K1570-01, Thermo Fisher, USA) according to the manufacturer's instructions. HiScript II Q RT SuperMix for qPCR (R222-01, Vazyme, Nanjing, China) was used to reverse-transcribe the mRNA/circRNA to DNA templates. miRNA was reverse-transcribed by using SuperScript™ III Reverse Transcriptase (18080085, Thermo Fisher, USA). Quantitative altimeter PCR was performed with PowerUp™ SYBR™ Green Master Mix (A25779, Applied Biosystems, USA) with the CFX Connect Real-Time System (BIO-RAD, USA). For the circRNA, specific divergent primers crossing the back-spliced junction were designed. All reactions were analyzed in triplicate. Housekeeping genes U6 and actin were used to normalize the analysis of miRNA and mRNA/circRNA, respectively. The relative miRNA/mRNA/circRNA expression levels were assessed using the 2*ΔΔ*CT method. All the primers used in this study are listed in Supplementary Table S1.

### 2.9. Flow Cytometry Assays

To estimate the apoptosis of pretreated cells, the Annexin V-FITC/PI apoptosis detection kit (KGA108, KeyGEN BioTECH, China) was used according to the manufacturer's instructions. Apoptosis was detected with a flow cytometer (Cytek, DXPSF13, USA).

For mitochondrial ROS analysis, H2DCFDA (65480, MCE, China) was used to detect mitochondrial ROS in the cells as the manufacturer's instruction described. In brief, cells were cultured with DCFH-DA at 37°C for 30 min. After the samples were washed three times with PBS, mitochondrial ROS was measured using a flow cytometer mentioned above.

### 2.10. Animal Model

The anterior cruciate ligament transaction (ACLT) model is a classic model in mimicking the condition of OA [16, 17]. Since circFAM160A2 manifested an anti-OA role in vitro, we proceeded to further investigate the potential role of circFAM160A2 in OA in vivo. In our study, twenty-four-week old male C57BL/6J (wild type) were divided into four groups and used for the experiments. The adeno-associated virus circFAM160A2 wt and mut were constructed and packaged by Hanbio (Shanghai, China). We injected adeno-associated virus-overexpression circFAM160A2 particles (AAV-circ) and adeno-associated virus-overexpression circFAM160A2 mut particles (AAV-mut) into the knee joints of mice with sham surgery or OA induced by ACLT surgery. After four weeks of treatment, mice were executed and treated as our previous study [18].

### 2.11. Statistical Analysis

All quantitative data are presented as the mean ± SDs. One-way ANOVA with subsequent post hoc Tukey's test was used for multiple comparisons. The value of *P* < 0.5 was considered to indicate significant differences.

## 3. Results

### 3.1. miR-505-3p Is Upregulated in OA Tissue and Directly Targets SIRT3

**Figure 1:**
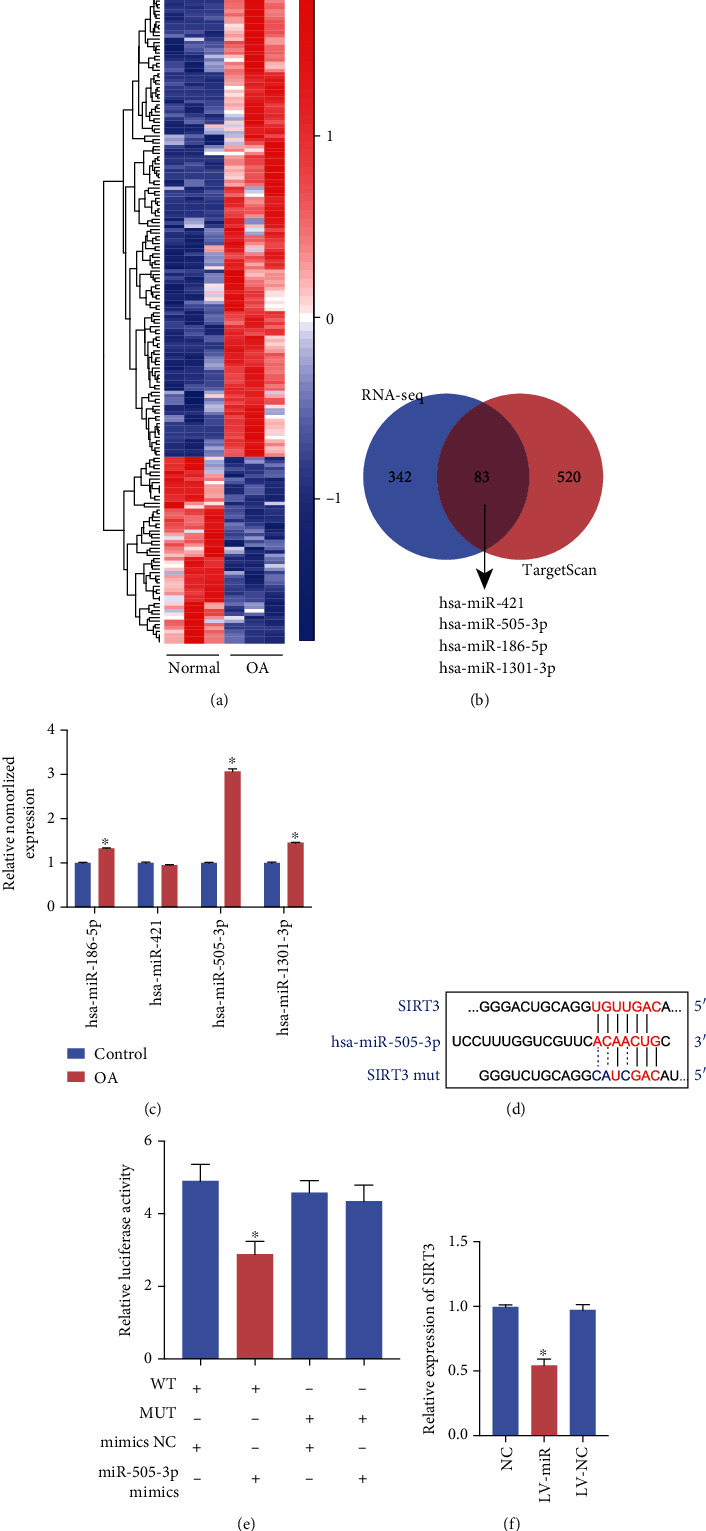
miR-505-3p specifically binds to SIRT3. (a) Heat map of all differentially expressed circular RNAs between OA and normal chondrocyte samples. *N* = 6 (three different samples in each group). (b) Schematic illustration to show the overlapping of the potential target miRNAs of SIRT3, as predicted by TargetScan and miRNAs upregulated in OA identified by sequencing analysis. (c) The relative levels of 4 miRNA candidates in the human articular chondrocytes were examined by qRT-PCR. (d) The potential binding sites of miR-505-3p and SIRT3. (e) Luciferase reporter assay was performed to detect the luciferase activities of HEK-293T cells cotransfected with a luciferase reporter construct containing wild-type or mutant SIRT3 and miR-505-3p mimics or mimics NC. (f) The relative expression of SIRT3 in human articular chondrocytes treated with lentivirus-miR-505-3p or lentivirus vector was examined by qRT-PCR (data are presented as the mean ± SD, ^∗^*P* < 0.05 vs. control or as indicated by Student's *t*-test).

The heat map of the microarray expression profile shows the differential expression of all miRNAs (Figure 1(a)). Four candidate miRNAs—hsa-miR-421, hsa-miR-505-3p, hsa-miR-186-5p, and hsa-miR-1301-3p—were selected from the TargetScan database according to their scores (Figure 1(b)). The expression levels of three miRNAs were significantly higher than those in the controls (Figure 1(c)). However, hsa-miR-505-3p had a higher ratio of expression in OA to expression in the control group than the other miRNAs and was consequently studied in subsequent experiments. The 568th to 575th bases of the SIRT3 3′ untranslated region (UTR) was specifically bound by miR-505-3p on an 8mer binding target site type, according to the database (Figure 1(d)). Reduced luciferase activity of the SIRT 3′-UTR was observed under miR-505-3p overexpression. In contrast, luciferase activity was much higher when a mutated form of SIRT3 3′-UTR was used (Figure 1(e)). Most efficient miRNAs function posttranscriptionally by base-pairing to the mRNA 3′-UTR to inhibit protein synthesis [19, 20]. The overexpression of miR-505-3p downregulated SIRT3 mRNA levels (Figure 1(f)).

### 3.2. Overexpression of miR-505-3p Contributes to ECM Degradation and Chondrocyte Apoptosis

**Figure 2:**
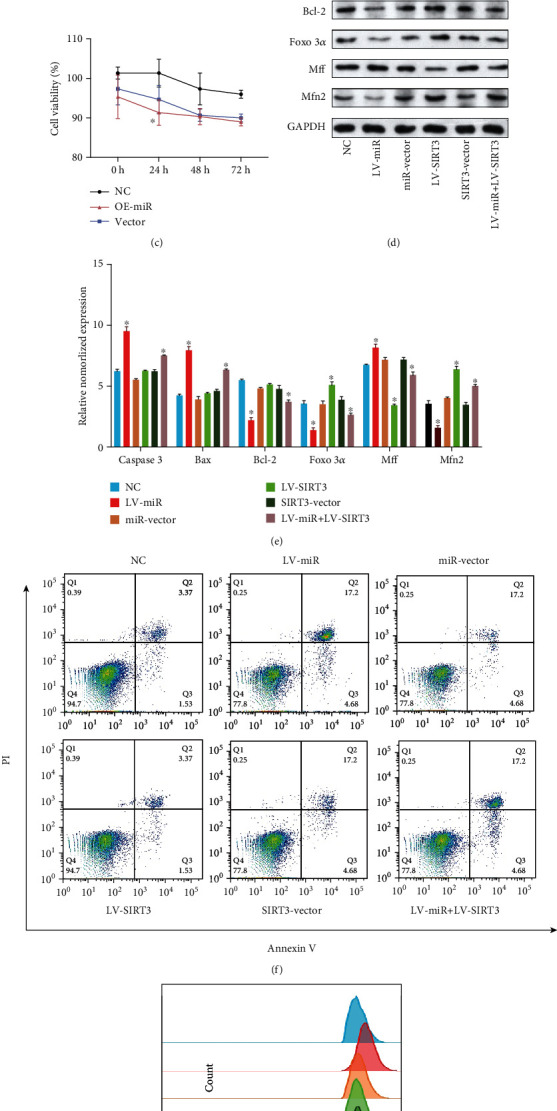
Overexpression of miR-505-3p induced chondrocyte apoptosis and mitochondrial dysfunction, while upregulating SIRT3 could reverse it. (a, b) The chondrocytes were transiently transduced with lentivirus-miR-505-3p or lentivirus vector, respectively. The levels of SIRT3, MMP3, COX2, COL2, and GAPDH were detected using WB (*N* = 3). (c) CCK-8 was performed to assess the viability of chondrocytes under the effect of miR-505-3p overexpression. (d, e) The chondrocytes were transduced with lentivirus-miR-505-3p, lentivirus vector, lentivirus-SIRT3 or lentivirus-miR-505-3p, and lentivirus-SIRT3. The protein levels of caspase 3, Bax, Bcl-2, FOXO3*α*, Mff, Mfn2, and GAPDH were detected using WB (*N* = 3). (f) The analysis of chondrocyte apoptosis was performed by flow cytometry. (g) Flow cytometry analysis of mitochondrial ROS level of each group. ^∗^*P* < 0.05 vs. control. Data are presented as the mean ± SD.

SIRT3 was also observed to be downregulated at the protein level in the LV-miR group compared with the control and lentivirus vector groups. After overexpression of miR-505-3p using LV-miR, both matrix metalloproteinase 3 (MMP3) and cyclooxygenase 2 (COX2) were overexpressed, while a reduction in chondrocyte-specific protein type II collagen (COL2) expression was observed relative to that in the control (Figures 2(a) and 2(b)). CCK-8 results indicated that under 24-hour treatment, the lentivirus vector inhibited chondrocyte viability down to 95% with no significance, whereas overexpression of miR-505-3p exacerbated the inhibitory effect and significantly decreased the chondrocyte viability to 91%. Collectively, these data suggest that overexpression of miR-505-3p accelerates OA progression in chondrocytes.

### 3.3. Upregulation of SIRT3 Rescues miR-505-3p-Induced Mitochondrial Dysfunction

In our previous review [21], we systematically elaborated the vital role of SIRT3 in mitochondrial homeostasis in OA. Since miR-505-3p was able to modulate SIRT3 expression, we considered that its role in mitochondrial metabolism and function was worthy of further research. A previous study showed that SIRT3 is able to protect mitochondria from oxidative damage by deacetylating forkhead box O3*α* (FOXO3*α*) [22]. The same elevated protein expression level of FOXO3*α* was observed in the present study (Figures 2(d) and 2(e)). Mitochondrial fission and fusion are competing processes contributing to changes in mitochondrial morphology and metabolism. Mitofusin 2 (MFN2) is a key regulator of mitochondrial fusion, while mitochondrial fission factor (MFF) is required for mitochondrial fission [23, 24]. Western blot results showed that overexpression of miR-505-3p upregulated MFF and downregulated MFN2, while SIRT3 overexpression reversed this effect (Figures 2(d) and 2(e)). The altered expression of MFF and MFN2 indicated that SIRT3 downregulation led to a shift in mitochondrial dynamics from fusion to fission. Mitochondrial fusion is negatively associated with reactive oxygen species (ROS) production [25], which is consistent with our subsequent ROS detection experiment using oxidized DCFDA and flow cytometry. Mitochondrial dysfunction occurs as a result of disruption of fusion, while ROS is the byproduct of normal mitochondrial metabolism and homeostasis [26]. Jones proposed that excessive levels of ROS cause cellular damage and subsequently contribute to the progression of age-related diseases, such as OA [27]. Overexpression of SIRT3 rescued the increase in ROS induced by overexpression of miR-505-3p (Figure 2(g)). These results support our hypothesis that upregulation of SIRT3 could rescue miR-505-3p-induced mitochondrial dysfunction.

### 3.4. Upregulation of SIRT3 Reverses miR-505-3p-Induced Chondrocyte Apoptosis

As chondrocytes are the major cell type forming cartilage, their gradual apoptosis is a hallmark of OA pathogenesis and progression [28]. As the apoptosis assessment results show (Figures 2(d) and 2(e)), in the LV-miR group, the expression levels of proapoptotic proteins caspase 3 and Bax were significantly increased, whereas the expression of antiapoptotic protein, Bcl-2, was markedly decreased, when compared to that in the control. Meanwhile, a larger number of apoptotic cells were detected in the LV-miR group than in the control (Figure 2(f)). All of these proapoptosis effects could be rescued by overexpression of SIRT3.

### 3.5. circFAM160A2 Functions as an Efficient miR-505-3p Sponge in OA

**Figure 3:**
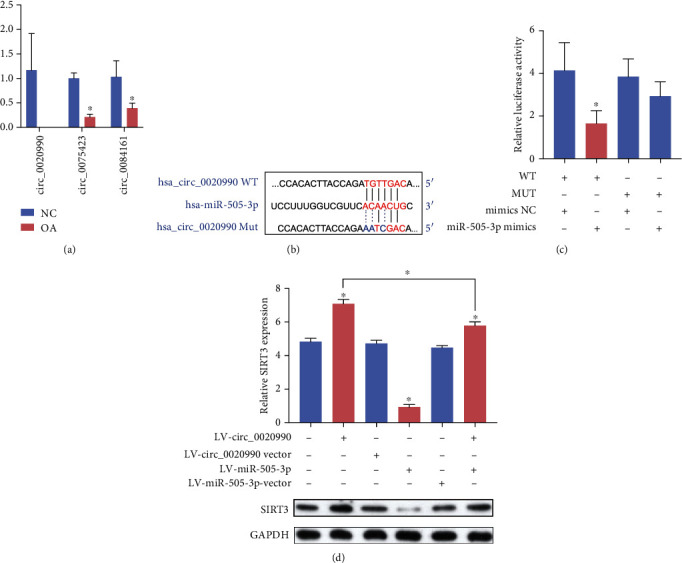
circFAM160A2 acts as a sponge of miR-505-3p. (a) The expression of hsa_circ_0020990, hsa_circ_0075423, and hsa_circ_0084161 was detected using qRT-PCR both in human OA cartilage and control cartilage tissue (*N* = 6). (b) The binding sites of miR-505-3p and circFAM160A2. (c) HEK-293T cells were transfected with miR-505-3p or NC and luciferase reporter constructs containing wt or mut circFAM160A2. (d) The chondrocytes were treated with or without lentivirus-circFAM160A2, lentivirus-miR-505-3p, or lentivirus vector, respectively. The levels of SIRT3 and GAPDH were detected using WB (*N* = 3) (data are presented as the mean ± SD, ^∗^*P* < 0.05 vs. control or as indicated by Student's *t*-test).

Recent studies have shown that several efficient circRNAs function as miRNA sponges to indirectly regulate the expression of target genes [13, 29]. miRanda analysis results are shown in Supplementary Table S2. The top three circRNAs, hsa_circ_0020990, hsa_circ_0075423, and hsa_circ_0084161, were selected for further investigation. Significant reductions in circRNA expression levels were observed in the OA tissues versus normal tissues for the three circRNAs. Interestingly, the expression of hsa_circ_0020990 in OA tissues was undetectable despite several optimization approaches (Figure 3(a)). Since its expression was detected in normal samples, it would not be unreasonable to assume that the actual expression of hsa_circ_0020990 simply falls below the RT-qPCR detection threshold. Therefore, hsa_circ_0020990 (circFAM160A2) was chosen for further study. The promising binding sites of circFAM160A2 (wt)-miR-505-3p are shown in Figure 3(b). We found that the luciferase activity of the miR-NC reporters was significantly higher than that of the miR-505-3p-transfected chondrocyte reporters. In contrast, the reporter containing the circFAM160A2 mut had the strongest luciferase activity, indicating that miR-505-3p can directly bind to circFAM160A2 (Figure 3(c)). To confirm the circFAM160A2-miR-505-3p-SIRT3 axis in chondrocytes, we design six groups: NC, LV-circFAM160A2, LV-circ vector, LV-miR-505-3p, LV-miR mut, and LV-circFAM160A2+LV-miR-505-3p. Western blot of SIRT3 results in circFAM160A2 overexpression models showing that overexpression of circFAM160A2 could upregulate the protein level of SIRT3, whereas using LV-miR to overexpress miR-505-3p would inhibit the impact of circFAM160A2 (Figure 3(d)). These results demonstrate that circFAM160A2 modulates the expression of SIRT3 by targeting miR-505-3p.

### 3.6. circFAM160A2 Attenuates OA-Related ECM Degradation and Chondrocyte Apoptosis In Vitro

**Figure 4:**
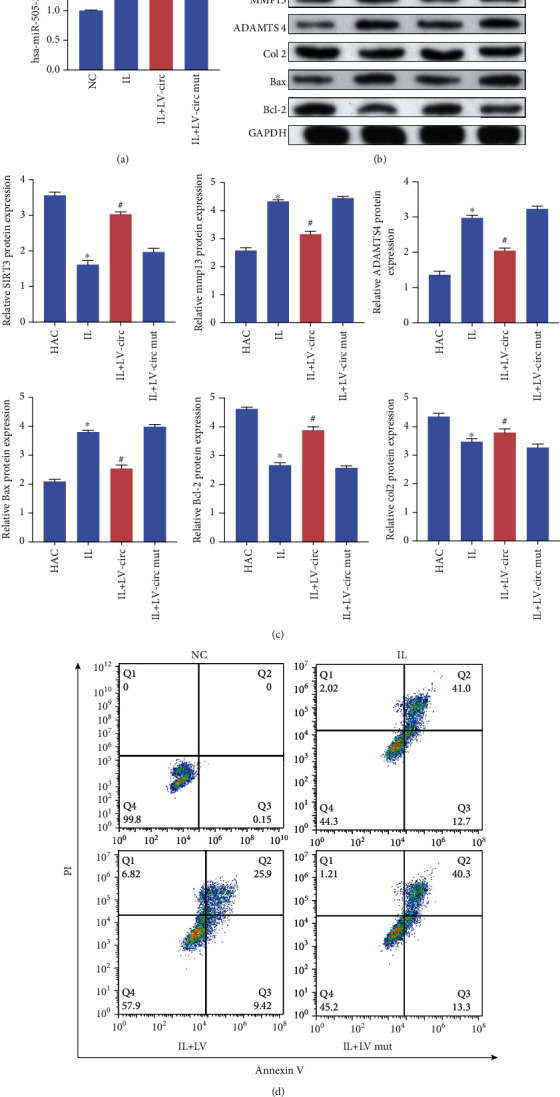
circFAM160A2 mediated ECM degradation and chondrocyte apoptosis in IL-1*β*-induced OA model. (a) The chondrocytes were treated with or without IL-1*β* and transduced with lentivirus-circFAM160A2 or mutant lentivirus-circFAM160A2. The relative expression of miR-505-3p was detected using qRT-PCR (*N* = 3). (b, c) The levels of SIRT3, MMP13, ADAMTS4, COL2, Max, Bcl-2, and GAPDH were detected using WB (*N* = 3). (d) Flow cytometry analysis of apoptosis level of each group (data are presented as the mean ± SD, ^∗^*P* < 0.05 vs. control, ^#^*P* < 0.05 vs. IL group by Student's *t*-test).

The miR-505-3p expression level was elevated in the IL-1*β* group, whereas using LV-circ significantly inhibited this elevation (Figure 4(a)). Meanwhile, compared with the IL-1*β* stimulated group, SIRT3, COL2, and Bcl-2 were significantly increased, while Bax and extracellular matrix (ECM) degrading associated proteins MMP13 and ADAMTS4 decreased in the LV-circ group (Figures 4(b) and 4(c)). A reduction in the number of apoptotic cells was also observed in the LV-circ group when compared to that in the control. Collectively, circFAM160A2 was demonstrated to play a positive role in ameliorating the progression of OA in vitro.

### 3.7. circFAM160A2 Alleviates OA Progression In Vivo

**Figure 5:**
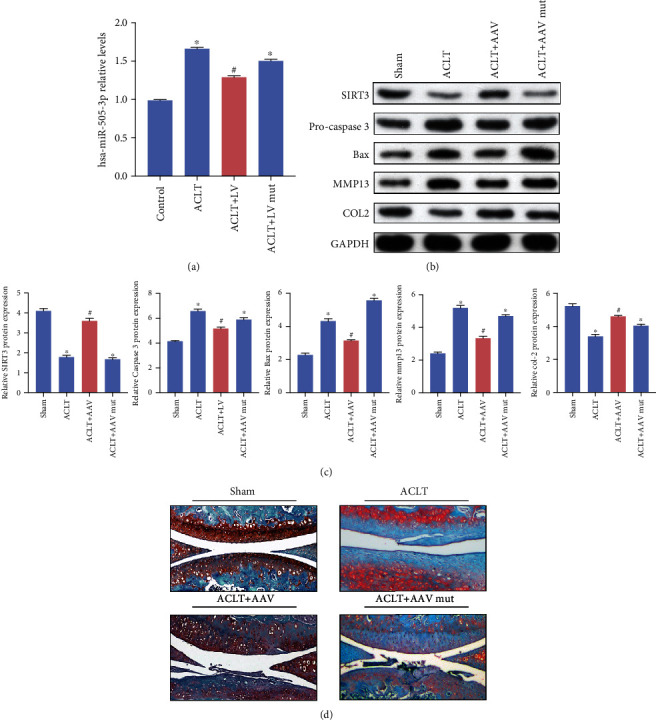
circFAM160A2 alleviates OA in vivo. (a) ACLT-induced OA mice were injected with AAV negative control, wt AAV circFAM160A2, or mut AAV circFAM160A2. The relative expression of miR-505-3p was detected using qRT-PCR (*N* = 3). (b, c) The relative expression of SIRT3, procaspase 3, Bax, MMP13, COL2, and GAPDH was detected using WB (*N* = 3). (d) Typical images of Safranin O/fast green staining of the cartilage in the indicated groups at six weeks after surgery (data are presented as the mean ± SD, ^∗^*P* < 0.05 vs. control, ^#^*P* < 0.05 vs. ACLT group by Student's *t*-test).

The effects of circFAM160A2 on miR-505-3p in vivo were similar to its in vitro effects (Figure 5(a)). The protein levels of MMP13 and proapoptotic proteins caspase 3 and Bax significantly decreased, while SIRT3 and COL2 markedly increased after treatment with circFAM160A2 (Figures 5(b) and 5(c)). Safranin O staining showed that compared to the AAV-mut group, the AAV-circ group had less cartilage destruction (Figure 5(d)). All of the in vivo results supported the protective role of circFAM160A2 in OA progression.

## 4. Discussion

OA is a prevalent degenerative joint disease whose etiology spans several disciplines, including biomechanics and biochemistry, and can involve multiple cellular and molecular pathways [30]. There have been some efforts to improve the understanding of OA causation and pathogenesis which may contribute to the identification of patients at greatest risk of disease and facilitate early diagnosis. However, for patients with clinical OA symptoms, most therapies are limited to pain management and joint replacement surgery. Until recently, no new therapeutic inquiries have been approved [2]. Elucidating the precise mechanism of OA progression for the development of improved therapies is a pressing and urgent task for researchers.

The sirtuin (SIRT) family, a class of evolutionarily conserved NAD^+^-dependent deacetylase proteins, contributes to the regulation of energy balance [7]. Among them, SIRT3 is a crucial member in regulating mitochondrial biogenesis and participates in diverse physiological and pathological processes. Recently, we found that SIRT3 could ameliorate osteoarthritis via regulating chondrocyte autophagy and apoptosis through the PI3K/Akt/mTOR pathway [37]. Besides, our previous study elaborated the vital role of SIRT3 in mitochondrial homeostasis in OA [21], and Wang et al. and Chen et al. further confirmed the protective role of SIRT3 in this condition [8, 9]. In the present study, diverse experiments collectively verified that SIRT3 ameliorated osteoarthritis progression via reducing chondrocyte apoptosis as well as improving mitochondrial dysfunction.

To further study the upstream regulating mechanism of SIRT3, we designed a series of experiments to determine the specific miRNA and circRNA that modulate the expression of SIRT3. Due to considerable progress in the field, the mechanisms by which miRNAs function as repressors of protein production, by inhibiting translation or destabilizing the mRNAs, are relatively well understood [31, 32]. The competitive endogenous RNA (ceRNA) hypothesis postulates that any RNA transcript that harbors miRNA-response elements (MRE) is able to sequester miRNAs from other targets sharing the same MREs [33], thus modulating related mRNA or protein expression. Recently, an increasing number of studies have indicated that circRNAs can act as ceRNAs via sponging miRNAs. Chen et al. reported that reference circRAPGEF5 could sponge miR-27a-3p, thereby inhibiting the growth and metastasis of renal cell carcinoma [34], while Sang et al. demonstrated that circRNA_0025202 could sponge miR-182-5p to regulate breast cancer progression [35]. In the OA research field, some researchers have also revealed that circRNAs act as miRNA sponges [13, 36]. However, most studies began with RNA-seq to identify differentially expressed circRNAs and subsequently investigated their binding miRNAs and potential roles in diseases. According to the ceRNA hypothesis, using databases and RNA-seq techniques to seek the miRNAs that inhibit a particular mRNA, and then finding the upstream circRNAs sponging the selected miRNA, is also a valid research strategy. Therefore, based on our previous work, we decided to investigate the potential miRNAs and circRNAs acting as ceRNA networks regulating SIRT3 directly or indirectly.

According to the ceRNA hypothesis, since SIRT3 is downregulated in OA, the miRNA and circRNA we looked for should be upregulated and downregulated, respectively. Thus, we used our upregulated miRNAs in OA from our RNA-seq results to combine with the TargetScan database to identify miRNAs that promisingly inhibit SIRT3 in OA. The top four candidate miRNAs were selected from the intersection. Based on their differential expression in OA compared with normal tissues and validated by the dual luciferase assay, miR-505-3p was chosen for further investigation. Subsequently, we confirmed that miR-505-3p could downregulate the expression of SIRT3 at both the mRNA and protein levels. We used lentivirus to establish a SIRT3 overexpression model and the miR-505-3p model to study their interaction. The results of western blotting, apoptotic cell detection, and ROS measurement using flow cytometry collectively indicated that miR-505-3p could aggravate the OA phenotype by promoting inflammation, ECM degradation, apoptosis, and mitochondrial dysfunction, whereas overexpression of SIRT3 could rescue this trend. Next, we used the miRanda database to find the circRNA sponging miR-505-3p. The top three circRNAs were selected for the RT-qPCR validation. Interestingly, we found that the expression of circFAM160A2 in OA tissues was too low to be detectable after we excluded the possible problems. The direct binding between circFAM160A2 and miR-505-3p was further validated using a dual luciferase assay. After a series of experiments as described above, circFAM160A2 upregulated the expression of SIRT3 by sponging miR-505-3p, thereby ameliorating the pathological process of OA in vitro.

In the mouse genome alignment, we found that one sequence shares 91.1% similarity with the circFAM160A2 sequence (1382 bp). Furthermore, the human and mouse FAM160A2 (3364 bp) sequence displayed up to 91.2% similarity. Additionally, miR-505-3p, SIRT3, and their binding sites are conserved in mice. A mouse ACLT-induced OA model was used for further investment in the effect of circFAM160A2 on OA progression in vivo. As mentioned above, we used adeno-associated virus to establish a circFAM160A2 overexpression model (AAV-circ). Subsequently, we injected the AAV-circ particles and AAV-mut particles into the knee joints of the mice under sham or ACLT surgery. Compared with the AAV-mut group, AAV-circ showed less expression of ECM degrading enzymes, less expression of proapoptotic proteins, and increased SIRT3 expression. Safranin O staining of the knee joint sections showed that the AAV-circ group exhibited less osteoarthritis characteristics than the AAV-mut group, with less cartilage loss. The results were consistent with the work we had performed in vitro, showing that circFAM160A2 could also exert its protective role in vivo.

In this study, we demonstrated that the circFAM160A2 promotes mitochondrial stabilization and apoptosis reduction in osteoarthritis chondrocytes by targeting miR-505-3p and SIRT3 using a series of experiments in vitro and in vivo. Unlike traditional circRNAs functioning as ceRNAs studies, we performed the study using the strategy of mRNAs to miRNAs and circRNAs. However, our work had several limitations. First, although the protective effects of circFAM160A2 on OA were demonstrated in our study, the possibility that other critical circRNAs involved in the progression of OA via modulating SIRT3 could not be excluded. Second, recent studies have shown the translational potential of circRNAs [15]. Whether circFAM160A2 affects OA progression by translating into proteins should be further considered.

In conclusion, our study demonstrated that circFAM160A2 can function as a ceRNA to sequester miR-505-3p, thereby upregulating the expression of SIRT3. Moreover, circFAM160A2 attenuates the progression of OA by stabilizing mitochondria and ameliorating apoptosis in osteoarthritis chondrocytes both in vitro and in vivo. This study suggests a potential therapeutic target for OA treatment.

## Data Availability

The datasets used and/or analyzed during the current study are available from the corresponding author on reasonable request.

## Supplementary Materials

Supplementary Materials All the primers used in this study are listed in Supplementary Table S1. miRanda analysis results of putative upstream targets of miR-505-3p are shown in Supplementary Table S2.
