# Supplementary material for: circFAM160A2 Promotes Mitochondrial Stabilization and Apoptosis Reduction in Osteoarthritis Chondrocytes by Targeting miR-505-3p and SIRT3
Source: Oxid Med Cell Longev. 2021 Oct 4;2021:5712280. doi: 10.1155/2021/5712280 (PMC8505077; doi:10.1155/2021/5712280)
Supplement: Supplementary Materials — All the primers used in this study are listed in Supplementary Table S1. miRanda analysis results of putative upstream targets of miR-505-3p are shown in Supplementary Table S2. [file 5712280.f1.doc]

Supplementary Table S1 Primers used in this study

| Primers for miRNA qPCR | | |
| --- | --- | --- |
| Human miR-505 | F | CGCGTCAACACTTGCTGG |
| Human miR-1301 | F | TTGCAGCTGCCTGGGAGT |
| Human miR-186 | F | CGCGCAAAGAATTCTCCTTTTG |
| Human miR-421 | F | GCGCGATCAACAGACATTAATT |
| U6 | F | CGCTTCGGCAGCACATATACTAA |
| micro-R | R | AGTGCAGGGTCCGAGGTATT |
| Primers for mRNA qPCR | | |
| actin | R | TGACGTGGACATCCGCAAAG |
|  | F | CTGGAAGGTGGACAGCGAGG |
| Sirt3 | R | GAGGCTCCTCCACGACAAG |
|  | F | GGGATCCCAGATGCTCTCTCA |
| Primers for circRNA qPCR | | |
| hsa_circ_0020990 | F | TGGCAGTAACTCCCGGATTTAG |
|  | R | GGTTTTCTCCACTTGTGTCTCCAG |
| hsa_circ_0075423 | F | GCCTGCCTCCAACAGGTAAC |
|  | R | AGGGACGCTGTCTGACTGAGA |
| hsa_circ_0084161 | F | AGCCTTCTAGATGATGCAAATCTAC |
|  | R | TAATGGCTTGGATGTACTCTATTCT |

| **circbase ID** | **length** | **miRNA D (miR_ID)** | **miRanda binding site (positions)** | **targetscan binding site (positions)** |
| --- | --- | --- | --- | --- |
| hsa_circ_0020990 | 1382 | hsa-miR-505-3p | 404 505 | 418 517 425 524 |
| hsa_circ_0075423 | 1188 | hsa-miR-503-3p | 619 | 491 632 496 639 |
| hsa_circ_0084161 | 1132 | hsa-miR-505-3p | 98 | 113 807 119 813 |
| hsa_circ_0123431 | 1967 | hsa-miR-505-3p | 88 | 101 248 107 253 |
| hsa_circ_123441 | 509 | hsa-miR-503-3p | 88 | 101 248 107 253 |
| hsa_circ_0124063 | 1821 | hsa-miR-505-3p | 144 | 156 824 162 830 |
| hsa_circ_0131939 | 1046 | hsa-miR-505-3p | 619 | 491 632 496 639 |
| hsa_circ_0020988 | 2448 | hsa-miR-503-3p | 404 505 | 418 517 425 524 |
| hsa_circ_0002665 | 343 | hsa-miR-505-3p | 162 | 174 180 |
| hsa_circ_0003441 | 454 | hsa-miR-505-3p | 341 | 355 362 |

Supplementary Table S2 Top 10 results of miRanda
